# Tuberculosis of the Bones and Joints

**Published:** 1897-12

**Authors:** D. L. Peeples

**Affiliations:** Vice-Pres., Navasota, Texas


For the Texas Medical Journal.

### TUBERCULOSIS OF THE BONES AND JOINTS.

BY D. L. PEEPLES, M. D., VICE-PRES., NAVASOTA, TEXAS.

Read at the meeting of the Brazos Valley Medical Society, November 10, 1897.

### DEFINITION.

Pulmonary and surgical tuberculosis are identically one and the same disease, distinguished only by the location and the tissues in which the tubercle bacillus is infixed for detrimental proliferation. Tuberculosis is a specific, general, local, infectious and inoculable disease, being only slightly contagious.

### HISTORY.

In the days of Hippocrates it was observed that phthisis resulted more or less as a sequela of various forms of traumatism, while the profession labored in total ignorance concerning its etiology and pathology. During the early part of the eighteenth century, physicians applied the following terms to tuberculosis: Necrosis, caries, scrofulosis, spina ventosa, tumor albus, osteopththoria, osterospongiosis, etc., of the bones and joints. The immaturity of our knowledge of tuberculosis within the last century, necessarily makes the history interesting, when we come to understand the modern train of thought in regard to the true nature and character of the disease. Within the last decade and a half the most startling revelations have resulted from the indomitable perseverence of the pathologist in bacteriological observations, and empirical research. Petit was continually perplexed, and understanding its intricacy, could not determine where to place these osseous lesions; whether to term them exostosis, caries, bone softening, or whether they were a group of separate bone diseases. Boerhove described tuberculosis as a destruction of the epiphyses, developing within and extending outward. In London, 1676, the first clinical description with any logical precision, depicting a tubercular joint was drawn by Wiseman, who designated this affection as tumor-albus-white swelling, but these terms are almost relegated to oblivion, and should be deservedly expurgated from surgical literature. So also have the terms scrofula and scrofulosis, been reprehensively expunged from medical nomenclature. In 1779 Benjamin Bell taught that tumor albus resulted from traumatism, or a scrofulosis diathesis. Later, the ingenious Laennec demonstrated the unicity of scrofulosis and tuberculosis. "Wein, in 1817, made a wide distinction between tumor albus proper, and scrofulous inflammation of joints." The first unicity of a morbid anatomical comparison in a case of tumor albus of the synovial membranes, with lesions of tuberculosis of the pulmonary organs, was described by Rokitansky in 1844. Virchow declared that most intrigue diseases of the joints were miliary tuberculosis, and Volkmann, in 1865, demonstrated beyond the possibility of a doubt the inoculability of tubercular products. However, the garb of darkness was thrown aside by the untiring efforts of the scientific eye and the great ingenuity of the immortal Robert Koch; thus, on the 24th day of March, 1882, the tubercle bacillus was, beyond question, evidently displayed to vision, triumphantly announced to the world by promulgation, and medical science stepped onward and upward in the elucidation of the pathology and etiology of tuberculosis, by the labors of this indisputably great man.

### HEREDITY.

Accepting the dogma that the tubercular bacillus is the exclusive cause of tuberculosis, regardless of what organ, emunctory or tissues in which such a condition may exist, we are compelled to abandon the theory of hereditary transmission. We do acknowledge a tuberculosis diathesis, which is merely an hereditary enfeeblement, a field for multiplication of the tubercular bacillus, a constitutional physical debility, a more fertile soil for the lodgment, development and proliferation of the micro-organisms. Roswell Park admits the non-inheritance of tubercular diseases. Jacobi says, "Hereditary transmission ought not to be presumed to exist at all, except in cases which occur at a very early period of life." Transmission by heredity is denounced as a folly by numerous critical pathologists. Virchow has not been able to find a single case of hereditary infection. He had a case of tubercular endometritis in which the foetus was delivered and found free from tubercles. Therefore, tuberculous heredity cannot be admitted, unless some abnormal placental circulation exists. "Congenital tuberculosis in man has not yet been observed with certainty, as far as I know, and consequently there is a tendency not to admit the possibility of an inheritance of the poison of tubercular disease, as of other hereditary infectious diseases."—Tillmann.

Thus we can no longer adhere to the doctrine of the inheritance of tuberculosis.

### MODE OF INVASION.

The tubercular bacillus is almost ever afloat in the atmospheric circulation, especially so in great and over-crowded cities, hence the most frequent portals of the human being, are the respiratory tracts, the surfaces of which may be inviting and prolific soil for its lodgment and development, then by multiplication, be mechanically forced into the pulmonary tissues, or again if unfavorable, be overcome by phagocytosis or resistability. The alimentary canal is also one of the avenues of ingress for the tubercular micro-organism, especially in children, conveyed through the medium of unsterilized tuberculous cow's milk, half cooked meats and other food products. The implantation of the tubercle bacillus may be witnessed in cutaneous abrasions, mucous membranes, etc. Subcutaneous infection will give rise to lymphatic expressions more pronouncedly, thus making way and becoming infiltrated in the lymph spaces of the cellular tissues, to become deposited into the lymphatic streams, and infecting the glands. Here the bacilli may remain for an indefinite period, but eventually passing the safety guards or glands and disseminating the parasitic micro-organism from gland to gland and finally is poured into the vascular system making complete circuits from time to time, apparently in a harmless manner. Here we find why trauma results in osseous tuberculosis. Strange, but true, tuberculosis of the bones is rarely seen in severe fractures and injuries even in turbercular diathesis, but in slight trauma, sufficient to produce hæmatophleboestasis, does it more frequently result. Tuberculosis of the bones and joints may occur at all ages, originating from traumatism, though generally confined to children on account of rapidly developing osseous tissues.

Chemiotaxis of Tuberculosis. Instead of rehashing the pathology, symtomatology, and prognosis of osseous tuberculosis, which is already most beautifully and exhaustively depicted in numerous text books, I am constrained to present in detail a few thoughts in regard to the modern idea of the chemiotactic powers of cell life. A lymphosite is merely a young leucocyte, therefore in dealing with cellula-vitae, we look to the activity of the mononuclear leucocytes, and the polynuclear corpuscles because they are the principal battle wagers against pathogenic bacteria. To illustrate: The mononuclear cells in man will not even attempt to attack the gonococcus or the streptococcus of erysipelas, while these two micro-organisms are bravely assaulted by the polynuclear neutrophile corpuscles, and on the other hand the bacillus of leprosy is unnoticed by the polynuclear cells, but instantly devoured by the mononuclear corpuscles. Of course we understand that the cell life alluded to possesses amoeboid movements, or migrative powers, which develop the attraction of both histological cells, and pathogenic bacteria to the field of war. The great army of phagocytes are marched through the vascular walls by the thoughtful provision of nature in establishing the phenomenal process known as diapedesis, for the protection of the animal economy. Tubercular foci of bone originating from traumatism, or any other cause quickly results in the abundant assembling of the mononuclear, and polynuclear leucocytes, that crowd to the infected area, as osteoplastic phagocytes for the purpose of englobing the tubercle bacilli, or what might be termed tubercular osteoclasts. It is under Pfeffer's positive chemiotactic law of attraction, and the reverse negative chemotaxis, in which a repugnance is displayed that we find necrotic bone or sequestræ in tuberculosis of the bones and joints. Sometimes bony sequestræ may undergo solution or remain indefinitely but completely surrounded by muriel calcifications established by the phenomenal cell workers in their defense of the parent organism. Again we see the non motile tubercle bacilli, that spy a store house filled with nutritive material (an infected area), and in their attempts to capture the spoils, so soon do we find the phagocytic cells on the battle fields, and we gaze up on the furore of war, sometimes resulting in a retreating foe, and sometimes in our repulsed friends, the leucocytes. Metchnikoff states that "Tubercle is composed of a collection of phagocytes, mesodermic in origin, which move towards the spot where the bacilli are situated and englobe them; therefore giant cells of tuberculosis probably originate by the transformation of the mononuclear globules which having been attracted to the bacilli as phagocytes and having spent their life's duty, or accumulating in untold numbers are again transformed into epithelioid giant cells, which then present thè characteristic pathological tubercle. Virulent bacteria it seems possess the power of excreting a kind of toxalbuminous substance which exercises a stupefying effect over the leucocytes, especially so a in tubercular diathesis, hence Bouchard's reasons for absence of chemiotactic powers of the leucocytes. Suppuration or cold abscess of tuberculosis of the osseous tissues, is the result of the death of the macrophages. The polynuclear leucocytes which devour the tubercle bacilli soon perish and are then gotton rid of by the mononuclear phagocytes. Each puss cell will be found on examination to present the lifeless body of a phagocyte, which died in the attempt to sustain the life of the parent organism. Not infrequently will the body of a dead phagocyte be found to contain several pathogenic bacteria. Therefore patheologists have proved the leucocytes to be the scavengers of our bodies, as definitely as we perceive buzzards to be the scavengers of this earth.

### TREATMENT.

On account of the enormity of the subject, and the deficiency of time, I am compelled to abandon the attempt to present any method or course to pursue in the treatment of surgical tuberculosis, except referring my hearers to the various late text books where the subject is fully covered.

